# (*Z*)-2-Benzyl­idenebenzo[*d*]thia­zolo[3,2-*a*]imidazol-3(2*H*)-one

**DOI:** 10.1107/S1600536812035003

**Published:** 2012-08-15

**Authors:** Hoong-Kun Fun, Ching Kheng Quah, Hatem A. Abdel-Aziz, Hazem A. Ghabbour

**Affiliations:** a X-ray Crystallography Unit, School of Physics, Universiti Sains Malaysia, 11800 USM, Penang, Malaysia; b Department of Pharmaceutical Chemistry, College of Pharmacy, King Saud University, PO Box 2457, Riyadh 11451, Saudi Arabia

## Abstract

The mol­ecule of the title compound, C_16_H_10_N_2_OS, is approximately planar, the dihedral angle between the 1,3-benzothia­zolo[3,2-*a*]imidazol-3(2*H*)-one and the benzyl­idene moieties being 4.10 (8)°. A weak intra­molecular C—H⋯S inter­action generates an *S*(6) ring. No inter­molecular hydrogen bonds are observed in the crystal structure.

## Related literature

For background to and the biological activity of thia­zolo[3,2-*a*]benzimidazoles, see: Al-Rashood & Abdel-Aziz (2010 ▶); Chimirri *et al.* (1988 ▶), For a related structure, references to our previous work in this area and references to further synthetic details, see: Fun *et al.* (2012 ▶).
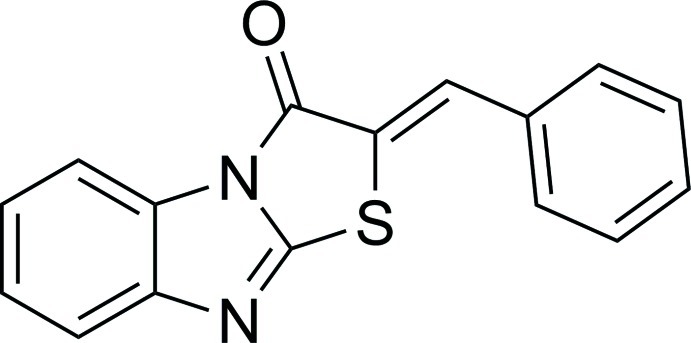


## Experimental

### 

#### Crystal data

C_16_H_10_N_2_OS
*M* *_r_* = 278.32
Orthorhombic, 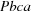

*a* = 12.1721 (5) Å
*b* = 7.7697 (3) Å
*c* = 27.2200 (8) Å
*V* = 2574.29 (16) Å^3^
*Z* = 8
Cu *K*α radiation
μ = 2.20 mm^−1^
*T* = 296 K
0.77 × 0.65 × 0.04 mm

#### Data collection

Bruker SMART APEXII CCD diffractometer
Absorption correction: multi-scan (*SADABS*; Bruker, 2009 ▶) *T*_min_ = 0.282, *T*_max_ = 0.917
8750 measured reflections
2270 independent reflections
1912 reflections with *I* > 2σ(*I*)
*R*_int_ = 0.051

#### Refinement

*R*[*F* ^2^ > 2σ(*F* ^2^)] = 0.046
*wR*(*F* ^2^) = 0.127
*S* = 1.03
2270 reflections
181 parameters
H-atom parameters constrained
Δρ_max_ = 0.45 e Å^−3^
Δρ_min_ = −0.20 e Å^−3^

### 

Data collection: *APEX2* (Bruker, 2009 ▶); cell refinement: *SAINT* (Bruker, 2009 ▶); data reduction: *SAINT*; program(s) used to solve structure: *SHELXTL* (Sheldrick, 2008 ▶); program(s) used to refine structure: *SHELXTL*; molecular graphics: *SHELXTL*; software used to prepare material for publication: *SHELXTL* and *PLATON* (Spek, 2009 ▶).

## Supplementary Material

Crystal structure: contains datablock(s) global, I. DOI: 10.1107/S1600536812035003/hb6910sup1.cif

Structure factors: contains datablock(s) I. DOI: 10.1107/S1600536812035003/hb6910Isup2.hkl

Supplementary material file. DOI: 10.1107/S1600536812035003/hb6910Isup3.cml

Additional supplementary materials:  crystallographic information; 3D view; checkCIF report

## Figures and Tables

**Table 1:** Hydrogen-bond geometry (Å, °)

*D*—H⋯*A*	*D*—H	H⋯*A*	*D*⋯*A*	*D*—H⋯*A*
C12—H12*A*⋯S1	0.93	2.56	3.262 (3)	132

**Fig. 1.**
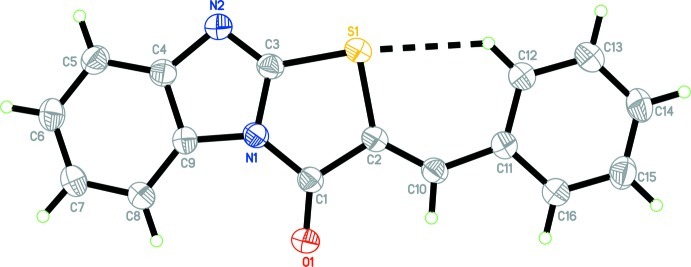
The molecular structure of the title compound showing 30% probability displacement ellipsoids for non-H atoms. Intramolecular hydrogen bond is shown as dashed line.

## supplementary crystallographic information

### Comment

Thiazolo[3,2-*a*]benzimidazoles show a variety of interesting biological activity such as antibacterial, antifungal, anti-inflammatory, antiulcer, antiviral, anthelmintic and anticancer activity (Al-Rashood & Abdel-Aziz, 2010; Chimirri *et al.*, 1988). As part on our ongoing studies in this area (Fun *et al.*, 2012), we now describe the crystal structure of the title compound.

In the title molecule, Fig. 1, the benzo[*d*]thiazolo[3,2-*a*]imidazol- 3(2*H*)-one moiety is roughly planar with an *r.m.s.* deviation 0.062 Å for the thirteen non H-atoms (C1–C9/N1/N2/O1/S1) and the benzilidene moiety is also nearly planar with an *r.m.s.* deviation 0.005Å for the seven non H-atoms (C10–C16). The dihedral between the two mean planes is 4.10 (8)°. An intramolecular C12—H12A···S1 weak interaction (Fig. 1 and Table 1) generates an S(6) ring, which helps to establish the planarity of the molecule. The bond distances are comparable to those in a related structure (Fun *et al.*, 2012). There are no significant intermolecular hydrogen bond observed in the crystal structure of this compound.

### Experimental

The one-pot synthesis of the title compound carried out by a cyclocondensation of 2-mercaptobenzimidazole, chloroacetic acid, benzaldehyde, acetic anhydride and glacial acetic acid in the presence of sodium acetate to afford the title compound (Fun *et al.*, 2012). Yellow plates were obtained by slowly evaporating an EtOH/DMF solution at room temperature.

### Refinement

All H atoms were positioned geometrically and allowed to ride on their parent atoms, with d(C—H) = 0.93 Å for aromatic and CH atoms, and the *U*_iso_(H) values were constrained to be 1.2*U*_eq_ of the carrier atoms.

### Crystal data

**Table:**

C_16_H_10_N_2_OS	*F*(000) = 1152
*M**_r_* = 278.32	*D*_x_ = 1.436 Mg m^−^^3^
Orthorhombic, *P**b**c**a*	Cu *K*α radiation, λ = 1.54178 Å
Hall symbol: -P 2ac 2ab	Cell parameters from 1434 reflections
*a* = 12.1721 (5) Å	θ = 3.3–69.8°
*b* = 7.7697 (3) Å	µ = 2.20 mm^−^^1^
*c* = 27.2200 (8) Å	*T* = 296 K
*V* = 2574.29 (16) Å^3^	Plate, yellow
*Z* = 8	0.77 × 0.65 × 0.04 mm

### Data collection

**Table:**

Bruker SMART APEXII CCD diffractometer	2270 independent reflections
Radiation source: fine-focus sealed tube	1912 reflections with *I* > 2σ(*I*)
Graphite monochromator	*R*_int_ = 0.051
φ and ω scans	θ_max_ = 67.4°, θ_min_ = 3.3°
Absorption correction: multi-scan (*SADABS*; Bruker, 2009)	*h* = −13→14
*T*_min_ = 0.282, *T*_max_ = 0.917	*k* = −8→6
8750 measured reflections	*l* = −32→28

### Refinement

**Table:**

Refinement on *F*^2^	Primary atom site location: structure-invariant direct methods
Least-squares matrix: full	Secondary atom site location: difference Fourier map
*R*[*F*^2^ > 2σ(*F*^2^)] = 0.046	Hydrogen site location: inferred from neighbouring sites
*wR*(*F*^2^) = 0.127	H-atom parameters constrained
*S* = 1.03	*w* = 1/[σ^2^(*F*_o_^2^) + (0.0558*P*)^2^ + 0.7634*P*] where *P* = (*F*_o_^2^ + 2*F*_c_^2^)/3
2270 reflections	(Δ/σ)_max_ = 0.001
181 parameters	Δρ_max_ = 0.45 e Å^−^^3^
0 restraints	Δρ_min_ = −0.20 e Å^−^^3^

### Special details

**Table:**

Geometry. All esds (except the esd in the dihedral angle between two l.s. planes) are estimated using the full covariance matrix. The cell esds are taken into account individually in the estimation of esds in distances, angles and torsion angles; correlations between esds in cell parameters are only used when they are defined by crystal symmetry. An approximate (isotropic) treatment of cell esds is used for estimating esds involving l.s. planes.
Refinement. Refinement of F^2^ against ALL reflections. The weighted R-factor wR and goodness of fit S are based on F^2^, conventional R-factors R are based on F, with F set to zero for negative F^2^. The threshold expression of F^2^ > 2sigma(F^2^) is used only for calculating R-factors(gt) etc. and is not relevant to the choice of reflections for refinement. R-factors based on F^2^ are statistically about twice as large as those based on F, and R- factors based on ALL data will be even larger.

### Fractional atomic coordinates and isotropic or equivalent isotropic displacement parameters (Å^2^)

**Table:**

	*x*	*y*	*z*	*U*_iso_*/*U*_eq_	
S1	0.78126 (4)	0.01576 (8)	0.554734 (19)	0.0518 (2)	
O1	1.04329 (12)	−0.1399 (2)	0.62205 (6)	0.0608 (5)	
N1	0.89265 (13)	0.0307 (2)	0.63592 (6)	0.0441 (4)	
N2	0.72939 (15)	0.1641 (3)	0.64488 (7)	0.0556 (5)	
C1	0.95869 (16)	−0.0762 (3)	0.60802 (7)	0.0450 (5)	
C2	0.90610 (16)	−0.0989 (3)	0.55886 (7)	0.0440 (5)	
C3	0.79414 (17)	0.0814 (3)	0.61580 (8)	0.0470 (5)	
C4	0.78928 (17)	0.1704 (3)	0.68946 (8)	0.0488 (5)	
C5	0.75984 (19)	0.2391 (3)	0.73449 (9)	0.0580 (6)	
H5A	0.6924	0.2931	0.7389	0.070*	
C6	0.8340 (2)	0.2249 (3)	0.77273 (8)	0.0591 (6)	
H6A	0.8156	0.2700	0.8033	0.071*	
C7	0.9350 (2)	0.1456 (3)	0.76682 (8)	0.0569 (6)	
H7A	0.9826	0.1387	0.7935	0.068*	
C8	0.96661 (18)	0.0763 (3)	0.72232 (8)	0.0506 (5)	
H8A	1.0349	0.0248	0.7180	0.061*	
C9	0.89105 (17)	0.0880 (3)	0.68454 (7)	0.0439 (5)	
C10	0.95483 (17)	−0.1957 (3)	0.52492 (8)	0.0472 (5)	
H10A	1.0213	−0.2430	0.5350	0.057*	
C11	0.92361 (16)	−0.2417 (3)	0.47492 (8)	0.0460 (5)	
C12	0.82753 (19)	−0.1866 (4)	0.45178 (9)	0.0592 (6)	
H12A	0.7789	−0.1147	0.4683	0.071*	
C13	0.8045 (2)	−0.2387 (4)	0.40425 (10)	0.0714 (7)	
H13A	0.7401	−0.2021	0.3892	0.086*	
C14	0.8757 (2)	−0.3439 (4)	0.37915 (9)	0.0659 (7)	
H14A	0.8594	−0.3784	0.3472	0.079*	
C15	0.9707 (2)	−0.3979 (4)	0.40123 (9)	0.0655 (7)	
H15A	1.0197	−0.4677	0.3842	0.079*	
C16	0.99336 (19)	−0.3485 (3)	0.44864 (8)	0.0554 (6)	
H16A	1.0574	−0.3879	0.4635	0.066*	

### Atomic displacement parameters (Å^2^)

**Table:**

	*U*^11^	*U*^22^	*U*^33^	*U*^12^	*U*^13^	*U*^23^
S1	0.0486 (4)	0.0588 (4)	0.0480 (3)	0.0084 (2)	−0.0101 (2)	−0.0030 (2)
O1	0.0484 (8)	0.0820 (12)	0.0522 (9)	0.0157 (8)	−0.0082 (7)	−0.0057 (8)
N1	0.0437 (9)	0.0443 (10)	0.0442 (9)	0.0009 (7)	−0.0052 (7)	−0.0007 (7)
N2	0.0549 (10)	0.0609 (13)	0.0509 (10)	0.0135 (9)	−0.0049 (8)	−0.0033 (9)
C1	0.0437 (11)	0.0476 (12)	0.0438 (11)	−0.0019 (9)	−0.0018 (9)	0.0002 (9)
C2	0.0416 (10)	0.0465 (13)	0.0438 (11)	−0.0023 (9)	−0.0034 (8)	0.0035 (9)
C3	0.0484 (11)	0.0451 (12)	0.0474 (11)	0.0040 (9)	−0.0060 (9)	0.0031 (9)
C4	0.0530 (12)	0.0463 (13)	0.0471 (11)	0.0022 (9)	−0.0013 (9)	0.0029 (9)
C5	0.0586 (13)	0.0613 (15)	0.0542 (13)	0.0076 (11)	0.0057 (11)	−0.0022 (11)
C6	0.0708 (14)	0.0632 (16)	0.0432 (12)	−0.0013 (12)	0.0060 (10)	−0.0032 (11)
C7	0.0656 (14)	0.0616 (15)	0.0437 (12)	−0.0062 (11)	−0.0053 (10)	−0.0001 (11)
C8	0.0499 (11)	0.0527 (14)	0.0492 (11)	−0.0013 (10)	−0.0059 (9)	0.0003 (10)
C9	0.0497 (11)	0.0409 (12)	0.0413 (10)	−0.0030 (9)	0.0006 (8)	0.0001 (9)
C10	0.0449 (10)	0.0520 (13)	0.0446 (11)	0.0002 (9)	−0.0022 (9)	0.0036 (10)
C11	0.0473 (10)	0.0461 (12)	0.0446 (11)	−0.0049 (9)	−0.0009 (9)	0.0016 (9)
C12	0.0542 (12)	0.0716 (17)	0.0519 (12)	0.0081 (11)	−0.0052 (10)	−0.0082 (12)
C13	0.0661 (15)	0.090 (2)	0.0578 (15)	0.0089 (14)	−0.0158 (12)	−0.0099 (14)
C14	0.0783 (16)	0.0715 (18)	0.0478 (12)	−0.0034 (13)	−0.0079 (12)	−0.0104 (12)
C15	0.0772 (16)	0.0656 (17)	0.0538 (13)	0.0069 (13)	0.0063 (12)	−0.0083 (12)
C16	0.0544 (13)	0.0606 (15)	0.0511 (13)	0.0049 (11)	−0.0014 (10)	0.0002 (11)

### Geometric parameters (Å, º)

**Table:**

S1—C3	1.746 (2)	C7—H7A	0.9300
S1—C2	1.765 (2)	C8—C9	1.383 (3)
O1—C1	1.205 (2)	C8—H8A	0.9300
N1—C3	1.376 (3)	C10—C11	1.457 (3)
N1—C1	1.383 (3)	C10—H10A	0.9300
N1—C9	1.396 (3)	C11—C16	1.386 (3)
N2—C3	1.289 (3)	C11—C12	1.396 (3)
N2—C4	1.417 (3)	C12—C13	1.384 (3)
C1—C2	1.494 (3)	C12—H12A	0.9300
C2—C10	1.331 (3)	C13—C14	1.373 (4)
C4—C5	1.384 (3)	C13—H13A	0.9300
C4—C9	1.401 (3)	C14—C15	1.369 (4)
C5—C6	1.382 (3)	C14—H14A	0.9300
C5—H5A	0.9300	C15—C16	1.374 (3)
C6—C7	1.384 (3)	C15—H15A	0.9300
C6—H6A	0.9300	C16—H16A	0.9300
C7—C8	1.380 (3)		
			
C3—S1—C2	90.54 (10)	C7—C8—H8A	121.9
C3—N1—C1	117.37 (17)	C9—C8—H8A	121.9
C3—N1—C9	105.91 (17)	C8—C9—N1	132.4 (2)
C1—N1—C9	136.04 (18)	C8—C9—C4	123.2 (2)
C3—N2—C4	103.21 (17)	N1—C9—C4	104.39 (17)
O1—C1—N1	124.72 (19)	C2—C10—C11	132.1 (2)
O1—C1—C2	127.0 (2)	C2—C10—H10A	113.9
N1—C1—C2	108.28 (17)	C11—C10—H10A	113.9
C10—C2—C1	119.84 (19)	C16—C11—C12	117.6 (2)
C10—C2—S1	128.66 (16)	C16—C11—C10	117.96 (19)
C1—C2—S1	111.49 (15)	C12—C11—C10	124.4 (2)
N2—C3—N1	115.54 (19)	C13—C12—C11	120.1 (2)
N2—C3—S1	132.50 (16)	C13—C12—H12A	119.9
N1—C3—S1	111.95 (15)	C11—C12—H12A	119.9
C5—C4—C9	119.3 (2)	C14—C13—C12	120.7 (2)
C5—C4—N2	129.7 (2)	C14—C13—H13A	119.6
C9—C4—N2	110.95 (18)	C12—C13—H13A	119.6
C6—C5—C4	117.8 (2)	C15—C14—C13	119.8 (2)
C6—C5—H5A	121.1	C15—C14—H14A	120.1
C4—C5—H5A	121.1	C13—C14—H14A	120.1
C5—C6—C7	121.9 (2)	C14—C15—C16	119.8 (2)
C5—C6—H6A	119.1	C14—C15—H15A	120.1
C7—C6—H6A	119.1	C16—C15—H15A	120.1
C8—C7—C6	121.6 (2)	C15—C16—C11	121.9 (2)
C8—C7—H7A	119.2	C15—C16—H16A	119.0
C6—C7—H7A	119.2	C11—C16—H16A	119.0
C7—C8—C9	116.2 (2)		
			
C3—N1—C1—O1	−174.8 (2)	C6—C7—C8—C9	−1.2 (4)
C9—N1—C1—O1	−5.8 (4)	C7—C8—C9—N1	−178.4 (2)
C3—N1—C1—C2	4.5 (3)	C7—C8—C9—C4	2.4 (3)
C9—N1—C1—C2	173.5 (2)	C3—N1—C9—C8	−179.5 (2)
O1—C1—C2—C10	−1.4 (4)	C1—N1—C9—C8	10.7 (4)
N1—C1—C2—C10	179.30 (19)	C3—N1—C9—C4	−0.1 (2)
O1—C1—C2—S1	179.0 (2)	C1—N1—C9—C4	−169.9 (2)
N1—C1—C2—S1	−0.3 (2)	C5—C4—C9—C8	−2.3 (3)
C3—S1—C2—C10	177.7 (2)	N2—C4—C9—C8	179.4 (2)
C3—S1—C2—C1	−2.71 (16)	C5—C4—C9—N1	178.3 (2)
C4—N2—C3—N1	−0.2 (3)	N2—C4—C9—N1	0.0 (2)
C4—N2—C3—S1	178.6 (2)	C1—C2—C10—C11	179.5 (2)
C1—N1—C3—N2	172.3 (2)	S1—C2—C10—C11	−1.0 (4)
C9—N1—C3—N2	0.2 (3)	C2—C10—C11—C16	−179.5 (2)
C1—N1—C3—S1	−6.8 (3)	C2—C10—C11—C12	−0.1 (4)
C9—N1—C3—S1	−178.84 (14)	C16—C11—C12—C13	0.1 (4)
C2—S1—C3—N2	−173.7 (3)	C10—C11—C12—C13	−179.3 (2)
C2—S1—C3—N1	5.20 (17)	C11—C12—C13—C14	−0.4 (5)
C3—N2—C4—C5	−178.0 (3)	C12—C13—C14—C15	−0.1 (5)
C3—N2—C4—C9	0.1 (3)	C13—C14—C15—C16	1.0 (4)
C9—C4—C5—C6	0.9 (4)	C14—C15—C16—C11	−1.3 (4)
N2—C4—C5—C6	178.8 (2)	C12—C11—C16—C15	0.8 (4)
C4—C5—C6—C7	0.2 (4)	C10—C11—C16—C15	−179.7 (2)
C5—C6—C7—C8	0.0 (4)		

### Hydrogen-bond geometry (Å, º)

**Table:**

*D*—H···*A*	*D*—H	H···*A*	*D*···*A*	*D*—H···*A*
C12—H12*A*···S1	0.93	2.56	3.262 (3)	132
